# Effects of an Isoquinoline Alkaloids Blend on the Expression of Genes Relevant for Antioxidant Capacity, Barrier Integrity and Inflammation Along the Broiler Gut

**DOI:** 10.1111/jpn.70012

**Published:** 2025-10-07

**Authors:** Vasileios V. Paraskeuas, Ioannis Brouklogiannis, Anja Pastor, Konstantinos C. Mountzouris

**Affiliations:** ^1^ Laboratory of Nutritional Physiology and Feeding, Department of Animal Science, School of Animal Biosciences Agricultural University of Athens Athens Greece; ^2^ Feed Innovations & Technologies P.C. A Spin‐Off Company of the Agricultural University of Athens Kifisia Greece; ^3^ Phytobiotics Futterzusatzstoffe GmbH, Eltville GermanyPhytobiotics Eltville Germany

**Keywords:** gut integrity, Nrf2 pathway, phytogenic, poultry, TLR signaling

## Abstract

Intensive broiler production may lead to perturbations of gut function and health. The maintenance of intestinal homeostasis in broilers through the use of dietary phytogenic components such as isoquinoline alkaloids (IQs) is an emerging topic of scientific investigations. In this respect, IQs effects on the underlying mechanisms involved in gut antioxidant capacity, barrier integrity and inflammatory response still remain unclear. This study aimed to investigate the effect of dietary administration of an IQs blend on the expression of genes relevant for gut antioxidant response, barrier function, and inflammatory status, along the intestine of 35‐days‐old broilers. One hundred eighty‐two one‐day‐old Ross 308 broilers were randomly distributed into 2 experimental treatments with 7 replicates of 13 broilers each for 35 d, namely: control treatment (NC) with no IQ addition in the diet and treatment (M) with dietary supplementation at 200 mg/kg diet of an IQs standardized blend. Broiler performance responses did not differ (*p* > 0.05) among the two treatments. The results showed that M birds had significantly higher (*p* < 0.05) expression levels for antioxidant capacity, barrier integrity and inflammation‐related genes, at the duodenum compared to NC. Furthermore, at the cecal level, treatment M showed significant (*p* < 0.05) downregulation of TLR (Toll‐like receptors) signaling and the subsequent inflammation components, compared to NC. Overall, under the optimal conditions of the trial, there were no significant growth performance benefits of IQs blend dietary addition in broilers diets. Nevertheless, IQs blend promoted gut homeostasis in 35‐days‐old broilers via the beneficial modulation of antioxidant and inflammatory responses, primarily at the duodenal level. The latter, under stress challenge conditions, may prove beneficial also for performance that needs to be specifically studied.

## 1 Introduction

In animals, gut health is essential for their general health status and their productivity. Gut health is defined as the absence and prevention of disease along the intestine so that the animal will have the ability to perform its physiological functions and to deal effectively with exogenous and endogenous stressors (Kogut and Arsenault 2016). In poultry industry, commercial broilers are constantly exposed to several noninfectious environmental factors such as nutrient imbalances, antinutritional factors, heat stress and mycotoxins that can act as gut health disruptors and cause oxidative stress, loss of barrier integrity and immune dysfunctions (Soares et al. 2023).

Although in recent years, several scientific findings on the topic of broilers gut health have been published, the complete understanding of the underlying regulation mechanisms and interactions is still unclear (Wickramasuriya et al. 2022; Mountzouris et al. 2022). Moreover, considering that animal nutrition and maintenance of gut health go hand in hand with the quality of meat products, phytogenics are gaining momentum as gut function and health modulators in broiler production (Liu et al. 2024; Mountzouris and Brouklogiannis 2024).

Isoquinoline alkaloids (IQs) are phytogenic components that are mainly extracted from the plant *Macleaya cordata* (Dong et al. 2020). *Macleaya cordata* extract contains bioactive compounds, such as sanguinarine and chelerythrine, that have been approved as dietary supplements in the EU from 2005, and are known to be used widely in broiler production (Kikusato et al. 2021). In recent studies, IQs have been reported to enhance the growth‐performance of the broilers (Kikusato et al. 2021; Khongthong et al. 2023) by increasing intestinal antioxidant status (Liu et al. 2024; Paraskeuas et al. 2024) and barrier function (Liu et al. 2022a; Song et al. 2023; Liu et al. 2024; Paraskeuas et al. 2024), as well as modulating gut inflammatory responses (Kikusato et al. 2021; Khongthong et al. 2023; Song et al. 2023; Liu et al. 2024). However, the dietary IQs supplementation effects on gut antioxidant response, barrier integrity and immune function have not been holistically studied along the broiler intestine.

Therefore, this study aimed to investigate the effects of an IQs blend on broilers’ growth performance and gut antioxidant responses, barrier function and health. For this purpose, we evaluated the expression levels of critical genes related to nuclear factor (erythroid‐derived 2)‐like 2 (Nrf2), Toll‐like receptors (TLRs) and nuclear factor kappaB (NF‐κB) signaling pathways, including gut integrity‐tight junctions.

## 2 Materials and Methods

### 2.1 Animal and Diets

The housing and management of the animals were in compliance with the current European Union Directive on the protection of animals used for scientific purposes (EC, 43 2007; EU, 63 2010; Council of the European Union 2007, 2010), and the experimental protocol was approved (No7/03032020) by the Bioethics Committee of the Agricultural University of Athens (AUA), Greece. One hundred eighty‐two male one‐day‐old Ross 308 broilers were vaccinated for Marek's disease, infectious bronchitis and Newcastle disease. Broilers were obtained from a commercial hatchery and randomly allocated to 2 experimental treatments with 7 replicates per treatment. Each replicate had 13 broilers. All experimental treatments received a maize‐soyabean meal basal diet with coccidiostat inclusion, in mash form, formulated according to Ross 308 nutrient requirements for the starter (1–10 d), grower (11–24 d) and finisher (25–35 d) growth phases. The ingredients and the chemical composition of the experimental diet are presented in Table 1. Broilers were allocated to two treatments: NC (basal diet—no other additions), and M (basal diet containing 200 ppm of a standardized blend of isoquinoline alkaloids, provided as Sangrovit Feed (Phytobiotics Futterzusatzstoffe GmbH, Germany). Each treatment replicate was assigned to a clean floor pen (1 m^2^), and birds were raised on rice‐hull litter. Birds had 24 h light during Day 1, then 23 h light and 1 h dark until Day 7, and from Day 8 to 10 the lighting program was set to 18 h light and 6 h dark to the end of the trial. Throughout the experiment diets and water were available ad libitum.

**Table 1:** Ingredient (g/kg) and calculated chemical composition (g/kg as fed) of the basal experimental diets.

Ingredients	Starter (1 to 10 d)	Grower (11 to 24 d)	Finisher (25 to 35 d)
Maize	522.2	538.6	574.5
Soybean meal (44%)	349.1	345.1	323.6
Soy protein concentrate^a^	50.0	25.0	0.0
Soy oil	34.6	30.0	35.6
Vegetable fat^b^	0.0	22.0	30.0
Limestone	12.7	11.5	10.4
Mono calcium phosphate	15.0	13.3	11.8
Salt (NaCl)	2.1	2.4	2.4
Sodium bicarbonate	2.2	1.8	1.9
l‐lysine‐HCL	2.7	1.8	1.8
DL‐methionine	3.8	3.2	2.9
l‐threonine	1.1	0.8	0.6
Vitamin premix^c^	2.0	2.0	2.0
Mineral premix^d^	2.0	2.0	2.0
Coccidiostat^e^	0.5	0.5	0.5
Calculated chemical composition			
AME_n_ (MJ/kg diet)^f^	12.6	13.0	13.4
Dry matter (%)	90.01	90.01	90.06
Crude protein (%)^g^	23.0 (23.4)	21.5 (21.6)	19.5 (19.3)
Ether extract (%)^g^	5.82 (5.62)	7.52 (7.49)	8.91 (8.71)
Crude fiber (%)	3.76	3.68	3.53
Lysine (g/kg)	14.4	12.9	11.6
TSAA (methionine + cysteine) (g/kg)	10.8	9.9	9.1
Threonine (g/kg)	9.7	8.8	7.8
Calcium (g/kg)	9.6	8.7	7.9
Available phosphorus (g/kg)	4.8	4.4	4.0
Sodium (g/kg)	1.6	1.6	1.6

^a^ Soy Protein concentrate with 530 g crude protein/kg (Alpha Soy 530, Agilia Europe, Skjernvej 42, DK‐6920, Videbaek, Denmark).

^b^ Lecithinised fat powder with 6% lecithin (nufat 99 L, Nuevo SA, Schimatari, Viotia, Greece).

^c^ The vitamin premix for the starter period (Rovimix 11 Bro Basic, DSM, Netherlands) provided per kg of diet: 3.6 mg retinol (Vit.A), 100 μg cholecalciferol (Vit.D_3_), 80 mg Vit.E, 9 mg Menadione (Vit.K_3_), 3 mg Thiamine,7 mg Riboflavin, 6 mg Pyridoxine, 25 μg Cyanocobalamin, 50 mg Nicotinic acid, 15 mg Pantothenic acid, 1.5 mg Folic acid, 150 μg Biotin. The vitamin premix for the grower and finisher period (Rovimix 12 Bro Basic, DSM, Netherlands) provided per kg of diet: 3.6 mg retinol (Vit.A), 75 μg cholecalciferol (Vit.D_3_), 50 mg Vit.E, 7 mg Menadione (Vit.K_3_), 3 mg Thiamine, 6 mg Riboflavin, 6 mg Pyridoxine, 25 μg Cyanocobalamin, 40 mg Nicotinic acid, 12 mg Pantothenic acid, 1.2 mg Folic acid, 150 μg Biotin.

^d^ The mineral (Rovimix Bro M, DSM, Netherlands) provided per kg of diet: 400 mg choline chloride, 250 μg Co, 1.5 mg I, 300 μg Se, 50 mg Fe, 130 mg Mn, 20 mg Cu and 100 mg Zn.

^e^ Maxiban G160, Elanco, Elli Lilly and Company, Clinton Laboratories, Clinton, Indiana, USA.

^f^ Nitrogen‐corrected apparent metabolizable energy (AMEn).

^g^ The values in the parentheses were analyzed while the others were calculated.

### 2.2 Growth Performance Responses

Body weight (BW), and feed intake (FI), were monitored at the end of each growth phase (i.e. 1–10, 11–24, 25–35 and overall, 1–35 d), and were used to calculate body weight gain (BWG) and feed conversion ratio (FCR). Mortality was also monitored daily during the experiment.

### 2.3 RNA Isolation and Reverse‐Transcription PCR

At 35 days, one broiler per pen was randomly euthanized, the intestinal segments (duodenum, jejunum, ileum and ceca) were sampled, longitudinally opened and the luminal digesta were removed. Afterwards, the segments were washed thoroughly in 10 mL ice cold PBS‐EDTA (10 mM) solution (pH = 7.2) and a small piece (about 70–100 mg) was cut off and placed in sterile eppendorf tubes. Eventually, the total RNA from the intestinal segments was extracted using the NucleoZOL Reagent (Macherey‐Nagel GmbH; Co. KG, Germany), according to the manufacturer's protocol. RNA quantity and quality were determined by spectrophotometry (NanoDrop‐1000, Thermo Fisher Scientific, Waltham, United Kingdom).

Treatment with DNAse ensured the removal from the RNA samples of any contaminating genomic DNA. Ten μg of RNA were resuspended with 1 unit of DNase I (M0303, New England Biolabs Inc, Ipswich, UK) and 10 μL of 10x DNAse buffer to a final volume of 100 µL with the addition of DEPC water, for 20 min at 37°C. Before the DNAse inactivation at 75°C for 10 min, EDTA was added to a final concentration of 5 mM to protect RNA from being degraded during enzyme inactivation. RNA integrity was checked by agarose gel electrophoresis.

For cDNA preparation, 500 ng of total RNA from each sample were reverse‐transcribed to cDNA by PrimeScript RT Reagent Kit (Perfect Real Time, Takara Bio Inc., Shiga‐Ken, Japan) according to the manufacturer's recommendations. All cDNAs were then stored at –20°C.

The *Gallus gallus* genes below were investigated: Glyceraldehyde 3‐phosphate dehydrogenase (*GAPDH*), actin beta (*ACTB*), nuclear factor (erythroid‐derived 2)‐like 2 (*Nrf2*), kelch like ECH associated protein 1 (*Keap1*), catalase (*CAT*), superoxide dismutase 1 *(SOD1*), glutathione peroxidase 2 (*GPX2*), glutathione peroxidase 7 (*GPX7*), glutathione S‐transferase‐α *(GST*), glutathione reductase (*GSR*), NAD(P)H quinone dehydrogenase 1 (*NQO1*), heme oxygenase 1 (*HMOX1*), peroxiredoxin 1 (*PRDX1*), zonula occludens‐1 (*ZO1*), zonula occludens‐2 (*ZO2*),\claudin‐1 (*CLDN1*), claudin‐2 *(CLDN2*), claudin‐5 (*CLDN5*), occluding (*OCLN*) and mucin‐2 (*MUC2*), Toll‐like receptor 2 family member B (*TLR2B*), Toll‐like receptor 3 (*TLR3*), Toll‐like receptor 4 (*TLR4*), myeloid differentiation primary response 88 (*MyD88*), nuclear factor kappa B subunit 1 (*NF‐κB1*), toll like receptor adaptor molecule 1 (*TRIF*), =interferon regulatory factor 3 (*IRF3*), interleukin‐1 (*IL1*), interleukin‐6 (*IL6*), interleukin‐8 (*IL8*), interferon‐beta (*IFNW*), transforming growth factor beta 1 (*TGF‐β1*), cyclooxygenase 2 (*COX2*), lipopolysaccharide induced TNF factor (*LITAF*) and inducible nitric oxide synthase (*iNOS*) by quantitative real‐time PCR. Suitable primers were designed using the GenBank sequences deposited on the National Center for Biotechnology Information and US National Library of Medicine (NCBI) shown in Table 2. Primers were checked using the PRIMER BLAST algorithm for *Gallus gallus* mRNA databases to ensure that there was a unique amplicon.

**Table 2:** Oligonucleotide primers used for gene expression of selected targets by quantitative real time PCR.

Target	Primer sequence (5′‐3′)	Annealing temperature (⁰C)	PCR product size (bp)	GenBank (NCBI reference sequence)
Reference genes				
*GAPDH*	F: ACTTTGGCATTGTGGAGGGT R: GGACGCTGGGATGATGTTCT	59.5	131	NM_204305.1
*ACTB*	F: CACAGATCATGTTTGAGACCTT R: CATCACAATACCAGTGGTACG	60	101	NM_205518.1
Nrf2 pathway				
*NRF2*	F: AGACGCTTTCTTCAGGGGTAG R: AAAAACTTCACGCCTTGCCC	60	285	NM_205117.1
*KEAP1*	F: GGTTACGATGGGACGGATCA R: CACGTAGATCTTGCCCTGGT	62	135	XM_025145847.1
*CAT*	F: ACCAAGTACTGCAAGGCGAA R: TGAGGGTTCCTCTTCTGGCT	60	245	NM_001031215
*SOD1*	F: AGGGGGTCATCCACTTCC R: CCCATTTGTGTTGTCTCCAA	60	122	NM_205064.1
*GPX2*	F: GAGCCCAACTTCACCCTGTT R: CTTCAGGTAGGCGAAGACGG	62	75	NM_001277854.1
*GPX7*	F: GGCTCGGTGTCGTTAGTTGT R: GCCCAAACTGATTGCATGGG	60	139	NM_001163245.1
*GST*	F: GCCTGACTTCAGTCCTTGGT R: CCACCGAATTGACTCCATCT	60	138	NM_001001776.1
*GSR*	F: GTGGATCCCCACAACCATGT R: CAGACATCACCGATGGCGTA	62	80	XM_015276627.1
*NQO1*	F: GAGCGAAGTTCAGCCCAGT R: ATGGCGTGGTTGAAAGAGGT	60.5	150	NM_001277619.1
*HMOX1*	F: ACACCCGCTATTTGGGAGAC R: GAACTTGGTGGCGTTGGAGA	62	134	NM_205344.1
*PRDX1*	F: CTGCTGGAGTGCGGATTGT R: GCTGTGGCAGTAAAATCAGGG	61	105	NM_001271932.1
Gut barrier integrity				
*ZO1*	F: CTTCAGGTGTTTCTCTTCCTCCTC R: CTGTGGTTTCATGGCTGGATC	59.5	131	XM_413773
*ZO2*	F: CGGCAGCTATCAGACCACTC R: CACAGACCAGCAAGCCTACAG	59.5	87	NM_204918
*CLDN1*	F: CTGATTGCTTCCAACCAG R: CAGGTCAAACAGAGGTACAAG	59.5	140	NM_001013611
*CLDN2*	F: CAAGGACCGAGTGGCAGTG R: TTTGATGGAGGGCTGAGGA	62	289	NM_001277622.1
*CLDN5*	F: CATCACTTCTCCTTCGTCAGC R: GCACAAAGATCTCCCAGGTC	59.5	111	NM_204201
*OCLN*	F: TCATCGCCTCCATCGTCTAC R: TCTTACTGCGCGTCTTCTGG	62	240	NM_205128.1
*MUC2*	F: GCTGATTGTCACTCACGCCTT R: ATCTGCCTGAATCACAGGTGC	62	442	XM_015274015.1
TLR signaling				
*TLR2*	F: CTTGGAGATCAGAGTTTGGA R: ATTTGGGAATTTGAGTGCTG	62	238	NM_001161650.1
*TLR3*	F: GCTTGGTTTGCTAGTTGGCT R: ACCGTGATATTTAGGCGGGG	59.5	93	NM_001011691.3
*TLR4*	F: GTCTCTCCTTCCTTACCTGCTGTTC R: AGGAGGAGAAAGACAGGGTAGGTG	64.5	187	NM_001030693.1
*MyD88*	F: AATGGACACTGAGCTCTGCC R: CAAACCCGATCTGTGGGACA	60	126	NM_001030962.3
*NF‐κΒ1*	F: GAAGGAATCGTACCGGGAACA R: CTCAGAGGGCCTTGTGACAGTAA	59	131	NM_205134.1
*TRIF*	F: TCAGCCATTCTCCGTCCTCTTC R: GGTCAGCAGAAGGATAAGGAAAGC	62	339	NM_001081506.1
*IRF3*	F: GAGGATCCGGCCAAATGGAA R: GCCAAATCGTGGTGGTTGAG	60	212	NM_205372.1
Inflammation				
*IL1B*	F:GCTCTACATGTCGTGTGTGATGAG R: TGTCGATGTCCCGCATGA	62	80	XM_046931582.1
*IL6*	F: AAATCCCTCCTCGCCAATCT R: CCCTCACGGTCTTCTCCATAAA	59	106	NM_204628.1
*IL8*	F: CAGGTGACACCCGGAAGAAA R: CTGAACGTGCCTGAGCCATA	61	117	NM_205018.1
*IFNW*	F: CCTCAACCAGATCCAGCATTAC R: CCCAGGTACAAGCACTGTAGTT	60.5	167	NM_001024836.1
*TGF‐β1*	F: GGTTATATGGCCAACTTCTGCAT R: CCCCGGGTTGTGTTGGT	60	102	JQ423909.1
*COX2*	F: CTGCTCCCTCCCATGTCAGA R: CACGTGAAGAATTCCGGTGTT	60	123	XM_046922435.1
*LITAF*	F: GAGCAGGGCTGACACGGAT R: GCACAAAAGAGCTGATGGCAG	60	149	NM_204267.1
*iNOS*	F: ATTGTGGAAGGACCGAGCTG R: CCTCGCACACGGTACTCATT	60	141	NM_204961.1

Abbreviations: *ACTB*, actin, beta (reference genes); *CAT*, catalase; *CLDN1*, claudin‐1; *CLDN2*, claudin‐2; *CLDN5*, claudin‐5; *COX2*, cyclooxygenase 2; F, forward; *GAPDH*, glyceraldehyde 3‐phosphate dehydrogenase; *GPX2*, glutathione peroxidase 2; *GPX7*, glutathione peroxidase 7; *GSR*, Glutathione reductase; *GST*, Glutathione S‐transferase‐α; *HMOX1*, heme oxygenase 1; *IFNW*, interferon‐beta; *IL1*, interleukin‐1; *IL6*, interleukin‐6; *IL8*, interleukin‐8; *iNOS*, inducible nitric oxide synthase; *IRF3*, interferon regulatory factor 3; *Keap1*, kelch like ECH associated protein 1; *LITAF*, lipopolysaccharide induced TNF factor; *MUC2*, mucin‐2; *MyD88*, myeloid differentiation primary response 88; *NF‐κB1*, nuclear factor kappa B subunit 1; *Nrf2*, nuclear factor (erythroid‐derived 2)‐like 2; *NQO1*, NAD(P)H quinone dehydrogenase 1; *OCLN*, occludin; *PRDX1*, Peroxiredoxin 1; R, reverse; *SOD1*, superoxide dismutase 1; *TGF‐β1*, transforming growth factor beta 1; *TLR2B*, Toll‐like receptor 2 family member B; *TLR3*, Toll‐like receptor 3; *TLR4*, Toll‐like receptor 4; *TRIF*, toll like receptor adaptor molecule 1; *ZO1*, zonula occludens‐1; *ZO2*, zonula occludens‐2.

Real‐time PCR was performed in 96 well microplates with a SaCycler‐96 Real‐Time PCR System (Sacace Biotechnologies s.r.l.) and FastGene IC Green 2 x qPCR universal mix (Nippon Genetics, Tokyo, Japan). Each reaction contained 12.5 ng RNA equivalents as well as 200 nmol/L of forward and reverse primers for each gene.

The reactions were incubated at 50°C for 2 min, 95°C for 2 min, followed by 40 cycles of 95°C for 15 s 59.5°C to 64.5°C (depending on the target gene) for 15 s, 72°C for 1 min. This was followed by a melt curve analysis to determine the reaction specificity. Each sample was measured in duplicates. Relative expression ratios were calculated according to (Pfaffl 2001) and were adapted for the multi‐reference genes normalization procedure according to (Hellemans et al. 2007) using *GAPDH* and *ACTB* as reference genes.

### 2.4 Statistical Analysis

The normality of the experimental data was checked using the Kolmogorov–Smirnov test and was found to be normally distributed. Statistical analysis was performed using independent sample *t*‐tests to compare treatments NC and M using the SPSS for Windows Statistical Package Program (SPSS Inc., Chicago, IL). Growth performance parameters were analyzed on a pen basis and relative gene expression data evaluation was based on individual broilers. Statistical significance was declared at *p* < 0.05.

## 3 Results

### 3.1 Growth Performance Responses

The broiler growth performance responses during the starter (1–10 d), grower (11 to 24 d), finisher (25–35 d) periods and overall (1–35 d) are presented in Table 3. No significant (*p* > 0.05) differences were found between the experimental treatments for BW, BWG, FI and FCR.

**Table 3:** Broiler growth performance responses during the trial (1–35d).

	Treatments^a^			
Components^b^	NC	M	SEM^c^	*p*‐value^d^
Starter (d 1 to 10)				
BW (g)	243.4	247.7	5.54	0.716
BWG (g)	195.2	199.2	5.46	0.717
FI (g)	251.3	261.9	13.70	0.104
FCR	1.29	1.32	0.069	0.125
Grower (d 11 to 24)				
BW (g)	1076.6	1106.2	28.05	0.198
BWG (g)	833.2	858.4	24.89	0.304
FI (g)	1032.1	1077.3	31.38	0.552
FCR	1.24	1.26	0.033	0.347
Finisher (d 25 to 35)				
BW (g)	2102.2	2190.1	55.04	0.478
BWG (g)	1025.6	1083.9	40.68	0.875
FI (g)	1560.8	1607.4	31.08	0.481
FCR	1.53	1.49	0.061	0.479
Overall (d 1 to 35)				
BWG (g)	2054.0	2141.5	55.00	0.478
FI (g)	2844.2	2946.6	55.14	0.612
FCR	1.39	1.38	0.038	0.943

^a^ NC (basal diet—no other additives) and M (basal diet + 200 ppm of a standardized blend of isoquinoline alkaloids, provided as Sangrovit Feed, Phytobiotics Futterzusatzstoffe GmbH, Germany). Data represent treatment means from n = 7 replicate floor pens per treatment.

^b^ Body Weight (BW), Body weight gain (BWG), feed intake (FI) and feed conversion ratio (FCR).

^c^ Pooled standard error of means.

^d^ Means within the same row with different superscripts (a, b) differ significantly (*p* < 0.05).

Relative gene expression of TLR signaling pathway and inflammation‐related genes along the broiler gut.

### 3.2 Duodenum

In the duodenum, the relative expression of genes related to the Nrf2 pathway (*Keap1, CAT, SOD, GPX7, GSR, NQO1, HMOX1, PRDX1*), gut barrier integrity (*ZO1, ZO2, CLDN5, OCLN, MUC2*), TLR signaling pathway (*TLR2b, TLR3, TLR4, MyD88, NF‐κΒ1, IRF3, TRIF*) and inflammation (*IL1B, IL6, IL8, IFN1, TGF‐β1, COX2, LITAF, iNOS*) are presented in Table 4. The inclusion of IQs blend in broiler diets (M), increased the relative expression of *GSR* (*p* = 0.003) and *HMOX1* (*p* = 0.003) compared to treatment NC. Moreover, the expression levels of *CLDN5* (*p* < 0.001), were higher in treatment M compared to NC. Finally, IQs blend supplementation in broiler diets, decreased the expression levels of *IL8* (*p* = 0.013), *LITAF* (*p* = 0.031) and *iNOS* (*p* = 0.028) compared to the control treatment (Table 4).

**Table 4:** Relative gene expression of Nrf2 pathway, gut barrier integrity, TLR signaling and inflammation related genes in the duodenum of 35 d old broilers.

Genes^a^	Treatments^b^		Statistics	
Duodenum	NC	M	SEM^c^	*p*‐value^d^
Nrf2 pathway				
* NRF2*	0.92	1.06	0.225	0.537
* KEAP1*	1.03	0.92	0.213	0.629
* CAT*	0.90	1.52	0.388	0.134
* SOD1*	0.82	1.14	0.220	0.176
* GPX2*	1.21	0.86	0.203	0.109
* GPX7*	1.14	1.16	0.285	0.941
* GST*	0.99	1.02	0.317	0.919
* GSR*	0.48^a^	2.02^b^	0.327	0.003
* NQO1*	0.91	1.01	0.176	0.554
* HMOX1*	0.79^a^	1.42^b^	0.172	0.003
* PRDX1*	0.89	1.21	0.221	0.169
Gut barrier integrity				
* ZO1*	1.06	0.89	0.172	0.335
* ZO2*	0.96	1.01	0.130	0.684
* CLDN1*	0.90	1.34	0.204	0.068
* CLDN2*	1.00	0.92	0.334	0.808
* CLDN5*	0.61^a^	1.18^b^	0.116	< 0.001
* OCLN*	1.06	0.91	0.184	0.423
* MUC2*	1.03	1.10	0.243	0.765
TLR signaling				
* TLR2b*	1.51	1.38	0.491	0.782
* TLR3*	1.02	0.90	0.222	0.604
* TLR4*	1.16	1.60	0.313	0.183
* MyD88*	1.11	1.13	0.368	0.964
* NF‐κΒ1*	1.54	1.11	0.302	0.177
* IRF3*	1.08	1.18	0.236	0.675
* TRIF*	1.11	1.00	0.203	0.592
Inflammation				
* IL1Β*	1.66	1.02	0.545	0.260
* IL6*	1.07	1.30	0.276	0.419
* IL8*	1.86^b^	0.82^a^	0.357	0.013
* IFNW*	0.95	1.48	0.261	0.067
* TGF‐β1*	1.25	1.27	0.385	0.968
* COX2*	1.23	0.92	0.268	0.266
* LITAF*	1.46^b^	1.03^a^	0.178	0.031
* iNOS*	2.04^b^	1.03^a^	0.403	0.028

^a^ Relative expression ratios of target genes were calculated according to Pfaffl (2001) adapted for the multi‐reference genes normalization procedure according to Hellemans et l. (2007) using glyceraldehyde 3‐phosphate dehydrogenase (*GAPDH*) and actin beta (*ACTB*) as reference genes. *CAT*, catalase; *CLDN1*, claudin‐1; *CLDN2*, claudin‐2; *CLDN5*, claudin‐5; *COX2*, cyclooxygenase 2; *GPX2*, glutathione peroxidase 2; *GPX7*, glutathione peroxidase 7; *GSR*, Glutathione reductase; *GST*, Glutathione S‐transferase‐α; *HMOX1*, heme oxygenase 1; *IFNW*, interferon‐beta; *IL1*, interleukin‐1; *IL6*, interleukin‐6; *IL8*, interleukin‐8; *iNOS*, inducible nitric oxide synthase; *IRF3*, interferon regulatory factor 3; *Keap1*, kelch like ECH associated protein 1; *LITAF*, lipopolysaccharide induced TNF factor; *MUC2*, mucin‐2; *MyD88*, myeloid differentiation primary response 88; *NF‐κB1*, nuclear factor kappa B subunit 1; *Nrf2*, nuclear factor (erythroid‐derived 2)‐like 2; *NQO1*, NAD(P)H quinone dehydrogenase 1; *OCLN*, occludin; *PRDX1*, Peroxiredoxin 1; *TGF‐β1*, transforming growth factor beta 1; *TLR2B*, Toll‐like receptor 2 family member B; *TLR3*, Toll‐like receptor 3; *TLR4*, Toll‐like receptor 4; *TRIF*, toll like receptor adaptor molecule 1; *SOD1*, superoxide dismutase 1; *ZO1*, zonula occludens‐1; *ZO2*, zonula occludens‐2.

^b^ NC (basal diet ‐ no other additives) and M (basal diet + 200 ppm of a standardized blend of isoquinoline alkaloids, provided as Sangrovit Feed, Phytobiotics Futterzusatzstoffe GmbH, Germany).

^c^ Pooled standard error of means.

^d^ Data represent treatment means for 7 broilers per treatment. Means within the same row with different superscripts (a, b) differ significantly (*p* ＜ 0.05).

### 3.3 Jejunum

In the jejunum, the relative expression levels of *Nrf2* (*p* = 0.035), *GSR* (*p* = 0.015), *NQO1* (*p* = 0.004) and *PRDX1* (*p* < 0.001) were downregulated by IQs blend addition, compared to NC treatment. Moreover, the relative expression levels of *MyD88* (*p* = 0.026) and *iNOS* (*p* = 0.026) were decreased in treatment M compared to the un‐supplemented NC treatment (Table 5).

**Table 5:** Relative gene expression of Nrf2 pathway, gut barrier integrity, TLR signaling and inflammation related genes in the jejunum of 35 d old broilers.

Genes^a^	Treatments^b^		Statistics	
Jejunum	NC	M	SEM^c^	*p*‐value^d^
Nrf2 pathway				
* NRF2*	1.38^b^	0.95^a^	0.181	0.035
* KEAP1*	1.12	0.93	0.157	0.249
* CAT*	1.24	0.90	0.230	0.164
* SOD1*	1.18	0.90	0.227	0.243
* GPX2*	1.48	0.92	0.278	0.071
* GPX7*	1.29	1.00	0.292	0.353
* GST*	1.00	0.87	0.241	0.592
* GSR*	1.52^b^	0.89^a^	0.220	0.015
* NQO1*	1.43^b^	0.76^a^	0.189	0.004
* HMOX1*	1.36	1.15	0.325	0.539
* PRDX1*	1.38^b^	0.96^a^	0.144	0.013
Gut barrier integrity				
* ZO1*	1.26	1.55	0.252	0.263
* ZO2*	0.99	1.15	0.164	0.343
* CLDN1*	1.88	1.24	0.377	0.115
* CLDN2*	1.08	1.35	0.436	0.543
* CLDN5*	1.45	1.31	0.253	0.593
* OCLN*	1.14	0.96	0.194	0.365
* MUC2*	0.94	0.94	0.235	0.986
TLR signaling				
* TLR2b*	1.09	1.19	0.280	0.731
* TLR3*	1.09	0.86	0.271	0.410
* TLR4*	1.39	1.15	0.347	0.507
* MyD88*	1.54^b^	0.85^a^	0.098	0.026
* NF‐κΒ1*	1.19	1.01	0.168	0.297
* IRF3*	1.02	0.86	0.225	0.482
* TRIF*	1.07	0.85	0.169	0.204
Inflammation				
* IL1Β*	0.93	0.84	0.339	0.783
* IL6*	1.36	1.06	0.308	0.350
* IL8*	1.45	1.18	0.517	0.600
* IFNW*	0.95	1.14	0.193	0.337
* TGF‐β1*	1.27	1.52	0.478	0.608
* COX2*	0.94	1.30	0.343	0.317
* LITAF*	1.03	1.57	0.431	0.249
iNOS	1.94^b^	1.15^a^	0.308	0.026

^a^ Relative expression ratios of target genes were calculated according to Pfaffl (2001) adapted for the multi‐reference genes normalization procedure according to Hellemans et al. (2007) using glyceraldehyde 3‐phosphate dehydrogenase (*GAPDH*) and actin beta (*ACTB*) as reference genes. *CAT*, catalase; *CLDN1*, claudin‐1; *CLDN2*, claudin‐2; *CLDN5*, claudin‐5; *COX2*, cyclooxygenase 2; *GPX2*, glutathione peroxidase 2; *GPX7*, glutathione peroxidase 7; *GSR*, Glutathione reductase; *GST*, Glutathione S‐transferase‐α; *HMOX1*, heme oxygenase 1; *IFNW*, interferon‐beta; *IL1*, interleukin‐1; *IL6*, interleukin‐6; *IL8*, interleukin‐8; *iNOS*, inducible nitric oxide synthase; *IRF3*, interferon regulatory factor 3; *Keap1*, kelch like ECH associated protein 1; *LITAF*, lipopolysaccharide induced TNF factor; *MUC2*, mucin‐2; *MyD88*, myeloid differentiation primary response 88; *NF‐κB1*, nuclear factor kappa B subunit 1; *Nrf2*, nuclear factor (erythroid‐derived 2)‐like 2; *NQO1*, NAD(P)H quinone dehydrogenase 1; *OCLN*, occludin; *PRDX1*, Peroxiredoxin 1; *TGF‐β1*, transforming growth factor beta 1; *TLR2B*, Toll‐like receptor 2 family member B; *TLR3*, Toll‐like receptor 3; *TLR4*, Toll‐like receptor 4; *TRIF*, toll like receptor adaptor molecule 1; *SOD1*, superoxide dismutase 1; *ZO1*, zonula occludens‐1; *ZO2*, zonula occludens‐2.

^b^ NC (basal diet ‐ no other additives) and M (basal diet + 200 ppm of a standardized blend of isoquinoline alkaloids, provided as Sangrovit Feed, Phytobiotics Futterzusatzstoffe GmbH, Germany).

^c^ Pooled standard error of means.

^d^ Data represent treatment means for 7 broilers per treatment. Means within the same row with different superscripts (a, b) differ significantly (*p* ＜ 0.05).

### 3.4 Ileum

In the ileum, the dietary supplementation of the IQs blend increased the relative expression levels of *SOD* (*p* = 0.043) and *GPX2* (*p* = 0.027) compared to treatment NC. However, the expression levels of *NQO1* (*p* = 0.007) were decreased by IQs blend dietary administration, compared to the control treatment. Regarding the gut integrity genes studied, only the expression levels of *CLDN2* (*p* = 0.030) were increased with the IQs inclusion compared to treatment NC (Table 6). At last, the inclusion of the IQs blend increased the expression levels of *COX2* (*p* = 0.016) in treatment M compared to treatment NC (Table 6).

**Table 6:** Relative gene expression of Nrf2 pathway, gut barrier integrity, TLR signaling and inflammation related genes in the ileum of 35 d old broilers.

Genes^a^	Treatments^b^		Statistics	
Ileum	NC	M	SEM^c^	*p*‐value^d^
Nrf2 pathway				
* NRF2*	1.14	0.93	0.288	0.388
* KEAP1*	1.04	1.13	0.150	0.530
* CAT*	1.31	1.04	0.217	0.230
* SOD1*	0.82^a^	1.09^b^	0.116	0.043
* GPX2*	0.75^a^	1.13^b^	0.151	0.027
* GPX7*	1.12	1.29	0.210	0.434
* GST*	1.10	1.19	0.169	0.615
* GSR*	1.16	1.15	0.182	0.932
* NQO1*	1.08^b^	0.81^a^	0.084	0.007
* HMOX1*	1.07	0.93	0.136	0.300
* PRDX1*	1.17	1.07	0.201	0.618
Gut barrier integrity				
* ZO1*	0.85	1.02	0.106	0.144
* ZO2*	1.06	0.99	0.187	0.715
* CLDN1*	1.05	1.07	0.145	0.923
* CLDN2*	0.78^a^	1.49^b^	0.290	0.030
* CLDN5*	1.15	1.09	0.161	0.703
* OCLN*	1.00	1.02	0.180	0.920
* MUC2*	1.09	0.95	0.694	0.139
TLR signaling				
* TLR2b*	1.49	1.65	0.254	0.529
* TLR3*	0.93	1.17	0.348	0.496
* TLR4*	1.17	1.23	0.129	0.650
* MyD88*	1.20	1.44	0.484	0.623
* NF‐κΒ1*	1.10	0.98	0.188	0.521
* IRF3*	0.82	1.29	0.514	0.388
* TRIF*	1.13	0.95	0.165	0.296
Inflammation				
* IL1Β*	1.45	0.99	0.433	0.305
* IL6*	1.29	0.95	0.220	0.144
* IL8*	1.39	1.48	0.349	0.791
* IFNW*	1.16	0.97	0.226	0.400
* TGF‐β1*	1.38	1.13	0.241	0.317
* COX2*	0.81^a^	1.42^b^	0.218	0.016
* LITAF*	1.10	1.17	0.304	0.833
* iNOS*	1.43	1.09	0.490	0.501

^a^ Relative expression ratios of target genes were calculated according to Pfaffl (2001) adapted for the multi‐reference genes normalization procedure according to Hellemans et al. (2007) using glyceraldehyde 3‐phosphate dehydrogenase (*GAPDH*) and actin beta (*ACTB*) as reference genes. *CAT*, catalase; *CLDN1*, claudin‐1; *CLDN2*, claudin‐2; *CLDN5*, claudin‐5; *COX2*, cyclooxygenase 2; *GPX2*, glutathione peroxidase 2; *GPX7*, glutathione peroxidase 7; *GSR*, Glutathione reductase; *GST*, Glutathione S‐transferase‐α; *HMOX1*, heme oxygenase 1; *IFNW*, interferon‐beta; *IL1*, interleukin‐1; *IL6*, interleukin‐6; *IL8*, interleukin‐8; *iNOS*, inducible nitric oxide synthase; *IRF3*, interferon regulatory factor 3; *Keap1*, kelch like ECH associated protein 1; *LITAF*, lipopolysaccharide induced TNF factor; *MUC2*, mucin‐2; *MyD88*, myeloid differentiation primary response 88; *NF‐κB1*, nuclear factor kappa B subunit 1; *Nrf2*, nuclear factor (erythroid‐derived 2)‐like 2; *NQO1*, NAD(P)H quinone dehydrogenase 1; *OCLN*, occludin; *PRDX1*, Peroxiredoxin 1; *TGF‐β1*, transforming growth factor beta 1; *TLR2B*, Toll‐like receptor 2 family member B; *TLR3*, Toll‐like receptor 3; *TLR4*, Toll‐like receptor 4; *TRIF*, toll like receptor adaptor molecule 1; *SOD1*, superoxide dismutase 1; *ZO1*, zonula occludens‐1; *ZO2*, zonula occludens‐2.

^b^ NC (basal diet ‐ no other additives) and M (basal diet + 200 ppm of a standardized blend of isoquinoline alkaloids, provided as Sangrovit Feed, Phytobiotics Futterzusatzstoffe GmbH, Germany).

^c^ Pooled standard error of means.

^d^ Data represent treatment means for 7 broilers per treatment. Means within the same row with different superscripts (a, b) differ significantly (*p* ＜ 0.05).

### 3.5 Ceca

In the ceca, the relative expression levels of *Keap1* (*p* = 0.013) were decreased with IQs addition compared to treatment NC. Moreover, the relative expression levels of gut barrier genes such as *ZO1* (*p* = 0.014) and *ZO2* (*p* = 0.011) were downregulated with IQs blend compared to the control treatment NC (Table 7). The relative expression levels of TLR signaling‐related genes *TLR2b* (*p* = 0.006) and *TLR4* (*p* = 0.022) were decreased with IQs blend addition in broiler diets compared to non‐supplemented treatment NC. Finally, the expression levels of inflammation‐related genes *IFNW* (*p* = 0.003), *TGF‐β1* (*p* = 0.039) and *iNOS* (*p* = 0.046) were decreased with IQs blend inclusion compared to the control treatment (Table 7).

**Table 7:** Relative gene expression of Nrf2 pathway, gut barrier integrity, TLR signaling and inflammation related genes in the ceca of 35 d old broilers.

Genes^a^	Treatments^b^		Statistics	
Ceca	NC	M	SEM^c^	*p*‐value^d^
Nrf2 pathway				
* NRF2*	1.20	1.00	0.175	0.269
* KEAP1*	1.16^b^	0.68^a^	0.162	0.013
* CAT*	1.03	1.12	0.252	0.761
* SOD1*	0.94	1.02	0.206	0.724
* GPX2*	1.12	0.99	0.228	0.576
* GPX7*	0.93	0.77	0.150	0.306
* GST*	1.12	0.89	0.172	0.214
* GSR*	1.23	1.35	0.166	0.505
* NQO1*	1.16	1.45	0.408	0.488
* HMOX1*	1.26	1.11	0.193	0.453
* PRDX1*	0.97	1.31	0.276	0.233
Gut barrier integrity				
* ZO1*	1.19^b^	0.81^a^	0.130	0.014
* ZO2*	1.31^b^	0.96^a^	0.116	0.011
* CLDN1*	1.32	0.94	0.218	0.107
* CLDN2*	1.17	1.13	0.149	0.778
* CLDN5*	1.35	0.95	0.240	0.124
* OCLN*	1.13	1.10	0.190	0.871
* MUC2*	1.46	1.28	0.335	0.600
TLR signaling				
* TLR2b*	2.87^b^	1.34^a^	0.457	0.006
* TLR3*	1.06	0.94	0.170	0.479
* TLR4*	1.85^b^	1.03^a^	0.313	0.022
* MyD88*	1.01	1.13	0.150	0.465
* NF‐κΒ1*	0.90	1.09	0.183	0.342
* IRF3*	1.40	0.97	0.383	0.285
* TRIF*	1.24	0.99	0.128	0.071
Inflammation				
* IL1Β*	1.25	0.76	0.257	0.076
* IL6*	1.05	0.90	0.207	0.491
* IL8*	1.32	0.86	0.431	0.307
* IFNW*	1.41^b^	0.70^a^	0.192	0.003
* TGF‐β1*	1.20^b^	0.59^a^	0.262	0.039
* COX2*	1.27	0.77	0.266	0.084
* LITAF*	1.48	1.71	0.537	0.667
* iNOS* ^*5*^	2.63^b^	0.75^a^	0.843	0.046

^a^ Relative expression ratios of target genes were calculated according to Pfaffl (2001) adapted for the multi‐reference genes normalization procedure according to Hellemans et al. (2007) using glyceraldehyde 3‐phosphate dehydrogenase (*GAPDH*) and actin beta (*ACTB*) as reference genes. *CAT*, catalase; *CLDN1*, claudin‐1; *CLDN2*, claudin‐2; *CLDN5*, claudin‐5; *COX2*, cyclooxygenase 2; *GPX2*, glutathione peroxidase 2; *GPX7*, glutathione peroxidase 7; *GSR*, Glutathione reductase; *GST*, Glutathione S‐transferase‐α; *HMOX1*, heme oxygenase 1; *IFNW*, interferon‐beta; *IL1*, interleukin‐1; *IL6*, interleukin‐6; *IL8*, interleukin‐8; *iNOS*, inducible nitric oxide synthase; *IRF3*, interferon regulatory factor 3; *Keap1*, kelch like ECH associated protein 1; *LITAF*, lipopolysaccharide induced TNF factor; *MUC2*, mucin‐2; *MyD88*, myeloid differentiation primary response 88; *NF‐κB1*, nuclear factor kappa B subunit 1; *Nrf2*, nuclear factor (erythroid‐derived 2)‐like 2; *NQO1*, NAD(P)H quinone dehydrogenase 1; *OCLN*, occludin; *PRDX1*, Peroxiredoxin 1; *TGF‐β1*, transforming growth factor beta 1; *TLR2B*, Toll‐like receptor 2 family member B; *TLR3*, Toll‐like receptor 3; *TLR4*, Toll‐like receptor 4; *TRIF*, toll like receptor adaptor molecule 1; *SOD1*, superoxide dismutase 1; *ZO1*, zonula occludens‐1; *ZO2*, zonula occludens‐2.

^b^ NC (basal diet ‐ no other additives) and M (basal diet + 200 ppm of a standardized blend of isoquinoline alkaloids, provided as Sangrovit Feed, Phytobiotics Futterzusatzstoffe GmbH, Germany).

^c^ Pooled standard error of means.

^d^ Data represent treatment means for 7 broilers per treatment. Means within the same row with different superscripts (a, b) differ significantly (*p* ＜ 0.05).

## 4 Discussion

In poultry production, there is a wide range of factors that can undermine gut function and health, negatively affect nutrients digestion and thus compromise birds’ overall health and performance. Among them, the extensive exposure of broilers to several noninfectious dietary and environmental factors can result to oxidative stress, gut barrier integrity disruption and gut immune dysregulations (Dal Pont et al. 2021; Soares et al. 2023). Fortunately, the consequences of intensive broiler production on broiler intestinal health and performance can be attenuated by effective dietary strategies.

In this sense, phytogenic feed additives have been proven to exert positive effects on broiler gut antioxidant status (Song et al. 2018; Mountzouris et al. 2020), gut barrier function (Du et al. 2016; Paraskeuas and Mountzouris 2019; Pham et al. 2022), inflammatory response (Lee et al. 2017; Mountzouris et al. 2020) and the overall broiler growth performance (Hu et al. 2023; Zhang et al. 2023).

In particular, IQs derived from the plant *Macleaya cordata* have been shown in broilers to enhance the antioxidant status (Liu et al. 2024; Paraskeuas et al. 2024) and gut barrier integrity (Khongthong et al. 2023; Song et al. 2023; Paraskeuas et al. 2024), while reducing the inflammatory response (Kikusato et al. 2021; Khongthong et al. 2023; Song et al. 2023; Paraskeuas et al. 2024), resulting in improved overall gut health and productivity (Kikusato et al. 2021; Khongthong et al. 2023; Insawake et al. 2025).

Recently, in previous studies of the present research group have shown that the IQs blend application to broilers during the starter phase (1‐10 d) enhanced the antioxidant response along the intestine and beneficially modulated gut barrier integrity mainly at duodenal level (Paraskeuas et al. 2024). However, there is still lack of knowledge regarding the effects of IQs dietary supplementation on the metabolic pathways above including the inflammatory response along the broiler intestine at the end of the production cycle (e.g., 35 d). Therefore, the present study, in addition to antioxidant status and gut barrier integrity has explored further the expression of TLR members family and inflammation‐related genes along the intestine of finisher phase broilers.

TLRs are a wide family of vertebrate innate immunity, which is expressed in different tissues of chicken, including bursa, spleen, cecal tonsils and intestine and are responsible for recognizing conserved components of pathogens, typically known as pathogen associated molecular patterns (PAMPs) (Alizadeh et al. 2024). When TLRs bind to PAMPs, such as bacterial lipoproteins and peptidoglycans (*TLR2*), lipopolysaccharide (LPS) and glycolipids (*TLR4*) viral nucleic acids (*TLR3*), trigger signaling pathways including MyD88, TRIF and NF‐κΒ pathways, and subsequently initiate the secretion of a variety of cytokines, such as interleukin‐1 (*IL1*), interleukin‐6 (*IL6*), interleukin‐8 (*IL8*), interferon‐beta (*IFNW*), and other associated biomarkers that play important roles in broilers’ defense mechanisms (Wang et al. 2024).

Accumulating reports have demonstrated that IQs supplementation in broiler diets could decrease the expression of cytokines involved in inflammatory responses by reducing the expression of genes related to TLR and NF‐κB signaling pathways at the systemic and intestinal level. In particular, it has been evidenced that the supplementation of IQs in broiler diets downregulated liver *TLR4*, *MyD88*, *NF‐κB* mRNA expression levels (Liu et al. 2022b) and additionally reduced serum *IL6* and *TNF‐α* gene expression in laying hens (Guo et al. 2021). At intestinal level, IQs dietary addition downregulated jejunal mRNA levels of *NF‐κB*, *IL10* (Song et al. 2023) and the expression levels of *TLR2*, *TLR4* and *IL1*, *IL6*, *iNOS*, (Kikusato et al. 2021). In addition, IQs downregulated *NFκB* and *iNOS*, *IFN‐γ*, *IL4*, *TNF‐α*, in the ileum (Khongthong et al. 2023). In line with the above, the present study has also confirmed the beneficial modulation of inflammation response at the duodenum and cecal level.

**Figure 1:**
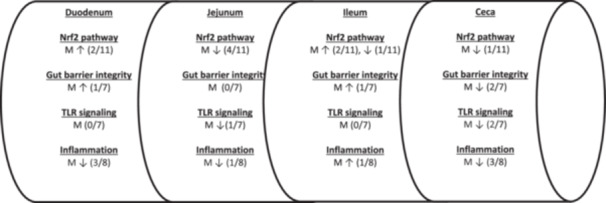
Summary of number and direction (↑↓) of changes shown for treatment M compared to treatment NC, regarding the Nrf2 pathway, gut barrier integrity, TLR signaling and inflammation related genes analyzed for their relative gene expression along the intestine of broilers at 35 d of age.

Regarding the broiler performance responses BW, BWG, FI and FCR, these did not reach statistical significance in this study. Similarly, in the work by (Wang et al. 2022), the dietary administration of a *Macleaya cordata* extract in broiler diets at the concentration of 1000 mg/kg did not improve growth performance indices at all the experimental periods. However, in the studies of Kikusato et al. (2021) and Khongthong et al. (2023) IQs supplementation at 100 mg/kg diet in both cases had positive effects on broiler BW, BWG, FI and FCR mainly at the grower to finisher growth periods and overall. The improvements in performance were explained by IQs antioxidant, immunomodulating and antibacterial properties (Kikusato et al. 2021; Khongthong et al. 2023). Variations in IQs dietary concentrations, their composition in bioactive substances, broiler genetics and breeding conditions between each experiment may explain the discrepancies between different studies (Paraskeuas et al. 2024). (Figure 1)

In this study, the dietary addition of IQs blend upregulated genes relative to antioxidant response (*GSR*, *HMOX1*), gut barrier integrity (*CLDN5*) and downregulated the expression of inflammation‐related genes such as *IL8*, *LITAF* and *iNOS* at the duodenum. Similarly to the results in this study, it has been shown in multiple instances with phytogenic applications that upregulation of the antioxidant response is concomitant with downregulation of the inflammatory response (Mountzouris et al. 2022). Regarding the expression levels of gut barrier integrity‐related genes, the *CLDN5* expression levels were upregulated by IQs blend supplementation at the duodenum. In the study of (Liu et al. 2024) the supplementation of *Macleaya cordata* IQs at concentration of 0.6 mg/kg of diet enhanced the antioxidant capacity in broiler small intestine via increasing the expression levels of genes related to Nrf2 signaling pathway (*GPX1*, *Nrf2*), while at the same time strengthened the gut barrier integrity by upregulating the *CLDN2* mRNA expression. Interestingly, the beneficial effect of IQs supplementation on the antioxidant status and gut barrier integrity biomarkers at duodenum was much more intense in young broilers, as it has been reported by a previous study of our research group (Paraskeuas et al. 2024). Collectively, the results reported highlight the overall cytoprotective potential of IQs and their contribution in maintaining gut barrier integrity and reducing the inflammatory response, mainly at duodenal level.

At the jejunal level, the relative expressions of the Nrf2 pathway‐related genes such as *Nrf2*, *GSR*, *NQO1*, *PRDX1* were reduced by IQs blend administration. Moreover, the downregulation of antioxidant response coincided with no further requirements for mRNA transcripts relative to gut barrier integrity, TLR signaling and inflammation indicating a balancing effect of IQs on jejunal homeostasis.

Cyclooxygenase‐2 (*COX2*) is a pro‐inflammatory enzyme that plays a vital role in intestinal inflammation and is needed for the maintenance of gut barrier integrity and homeostasis. It's increased expression in treatment M by 1.75 times compared to the control treatment was balanced by enhancements in the cytoprotective genes *SOD1* and *GPX2* as well as tight junction protein *CLDN2*. Generally, only if *COX2* exceeds multiple times it's baseline levels, could be considered as harmful to the intestinal barrier and cytoprotection status (Izadparast et al. 2022), which, given the above, was not the case here.

In the present study, at cecal level, IQs dietary supplementation significantly reduced mRNA expression levels of *TRL2* and *TLR4*, and also downregulated the expression of *IFNW*, *TGF‐β1* and *iNOS* molecules. These results were in line with studies which have shown that IQs could inhibit inflammatory response at intestinal level (e.g., jejunum, ileum) via reducing the expression of molecular biomarkers related to TLR and NF‐κΒ signaling pathways (Kikusato et al. 2021; Khongthong et al. 2023; Song et al. 2023). However, this is the first time that these findings were evidenced at the broilers’ ceca. In addition, the cecal analysis revealed that IQs supplementation resulted in downregulated TLR signaling and the consequent inflammatory response‐related genes possibly due to effects on gut microbiota (Paraskeuas and Mountzouris 2019). The above also justify the negative requirement for Nrf2 and tight junction mRNA transcripts.

## 5 Conclusion

In the present study, the IQs blend, enhanced cytoprotection mainly at the duodenum via increasing the expression levels of Nrf2 pathway‐related genes. The latter may have contributed to reduced requirements for mRNA transcripts of genes relative to antioxidant, gut barrier integrity and inflammatory responses downstream the duodenums. The powerful analytical nutrigenomic approach in this study generated new knowledge of IQs function along the gut of broilers at the finisher growth phase. The knowledge gained needs to be further evaluated under various environmental and nutritional challenges.

## Data Availability

The data that support the findings of this study are available from the corresponding author upon reasonable request.

## References

[jpn70012-bib-0001] Alizadeh, M. , B. Shojadoost , C. Fletcher , A. Wang , K. Abdelaziz , and S. Sharif . 2024. “Treatment of Chickens With Lactobacilli Prior to Challenge With *Clostridium perfringens* Modifies Innate Responses and Gut Morphology.” Research in Veterinary Science 172: 105241. 10.1016/j.rvsc.2024.105241. 38555776

[jpn70012-bib-0002] Dal Pont, G. C. , B. L. Belote , A. Lee , et al. 2021. “Novel Models for Chronic Intestinal Inflammation in Chickens: Intestinal Inflammation Pattern and Biomarkers.” Frontiers in Immunology 12: 676628. 10.3389/fimmu.2021.676628. 34054868 PMC8158159

[jpn70012-bib-0003] Dong, Y. , J. Lei , and B. Zhang . 2020. “Effects of Dietary Quercetin on the Antioxidative Status and Cecal Microbiota in Broiler Chickens Fed With Oxidized Oil.” Poultry Science 99: 4892–4903. 10.1016/j.psj.2020.06.028. PMC7598137 32988526

[jpn70012-bib-0004] Du, E. , W. Wang , L. Gan , Z. Li , S. Guo , and Y. Guo . 2016. “Effects of Thymol and Carvacrol Supplementation on Intestinal Integrity and Immune Responses of Broiler Chickens Challenged With *Clostridium perfringens* .” Journal of Animal Science and Biotechnology 7: 19. 10.1186/s40104-016-0079-7. 27006768 PMC4802587

[jpn70012-bib-0005] EC, 43 . 2007. “Council Directive of 28 June 2007 Laying down Minimum Rules for the Protection of Chickens Kept for Meat Production.” Official Journal of the European Union 182: 19–28.

[jpn70012-bib-0006] EU, 63 . 2010. “Directive of the European Parliament and of the Council of 22 September 2010 on the Protection of Animals Used for Scientific Purposes.” Official Journal of the European Union 276: 33–79.

[jpn70012-bib-0007] Guo, L. , J. Lv , Y. Liu , et al. 2021. “Effects of Different Fermented Feeds on Production Performance, Cecal Microorganisms, and Intestinal Immunity of Laying Hens.” Animals: An Open Access Journal From MDPI 11: 2799. 10.3390/ani11102799. 34679821 PMC8532698

[jpn70012-bib-0008] Hellemans, J. , G. Mortier , A. De Paepe , F. Speleman , and J. Vandesompele . 2007. “qBase Relative Quantification Framework and Software for Management and Automated Analysis of Real‐Time Quantitative PCR Data.” Genome Biology 8: R19. 10.1186/gb-2007-8-2-r19. 17291332 PMC1852402

[jpn70012-bib-0009] Hu, Z. , L. Liu , F. Guo , et al. 2023. “Dietary Supplemental Coated Essential Oils and Organic Acids Mixture Improves Growth Performance and Gut Health Along With Reduces Salmonella Load of Broiler Chickens Infected With Salmonella Enteritidis.” Journal of Animal Science and Biotechnology 14: 95. 10.1186/s40104-023-00889-2. 37391807 PMC10314490

[jpn70012-bib-0010] Insawake, K. , T. Songserm , O. Songserm , et al. 2025. “Effects of Isoquinoline Alkaloids as an Alternative to Antibiotic on Oxidative Stress, Inflammatory Status, and Cecal Microbiome of Broilers Under High Stocking Density.” Poultry Science 104: 104671. 10.1016/j.psj.2024.104671. PMC11719387 39689480

[jpn70012-bib-0011] Izadparast, F. , B. Riahi‐Zajani , F. Yarmohammadi , A. W. Hayes , and G. Karimi . 2022. “Protective Effect of Berberine Against LPS‐Induced Injury in the Intestine: A Review.” Cell Cycle 21: 2365–2378. 10.1080/15384101.2022.2100682. 35852392 PMC9645259

[jpn70012-bib-0012] Khongthong, S. , D. Faroongsarng , N. Roekngam , et al. 2023. “Sanguinarine‐Based Isoquinoline Alkaloids Modulated the Gut‐Brain Axis and Enhanced Growth Performance and Gut Integrity in Natural Heat Stress Broiler Chickens.” Livestock Science 275: 105297. 10.1016/j.livsci.2023.105297.

[jpn70012-bib-0013] Kikusato, M. , G. Xue , A. Pastor , T. A. Niewold , and M. Toyomizu . 2021. “Effects of Plant‐Derived Isoquinoline Alkaloids on Growth Performance and Intestinal Function of Broiler Chickens Under Heat Stress.” Poultry Science 100: 957–963. 10.1016/j.psj.2020.11.050. PMC7858171 33518149

[jpn70012-bib-0014] Kogut, M. H. , and R. J. Arsenault . 2016. “Editorial: Gut Health: The New Paradigm in Food Animal Production.” Frontiers in Veterinary Science 3: 346–358. 10.3389/fvets.2016.00071. PMC5005397 27630994

[jpn70012-bib-0015] Lee, M. T. , W. C. Lin , B. Yu , and T. T. Lee . 2017. “Antioxidant Capacity of Phytochemicals and Their Potential Effects on Oxidative Status in Animals—A Review.” Asian‐Australasian Journal of Animal Sciences 30: 299–308. 10.5713/ajas.16.0438. 27660026 PMC5337908

[jpn70012-bib-0016] Liu, Y. , K. Han , H. Liu , et al. 2024. “ *Macleaya cordata* Isoquinoline Alkaloids Attenuate *Escherichia coli* Lipopolysaccharide‐Induced Intestinal Epithelium Injury in Broiler Chickens by Co‐Regulating the TLR4/MyD88/NF‐κB and Nrf2 Signaling Pathways.” Frontiers in Immunology 14: 1335359. 10.3389/fimmu.2023.1335359. 38299145 PMC10828024

[jpn70012-bib-0017] Liu, Y. , Y. Li , J. Niu , et al. 2022b. “Effects of Dietary *Macleaya cordata* Extract Containing Isoquinoline Alkaloids Supplementation as an Alternative to Antibiotics in the Diets on Growth Performance and Liver Health of Broiler Chickens.” Frontiers in Veterinary Science 9: 950174. 10.3389/fvets.2022.950174. 35968000 PMC9363708

[jpn70012-bib-0018] Liu, Y. , Q. Wang , H. Liu , et al. 2022a. “Effects of Dietary Bopu Powder Supplementation on Intestinal Development and Microbiota in Broiler Chickens.” Frontiers in Microbiology 13: 1019130. 10.3389/fmicb.2022.1019130. 36312926 PMC9612830

[jpn70012-bib-0019] Mountzouris, K. C. , and I. Brouklogiannis . 2024. “Phytogenics as Natural Gut Health Management Tools for Sustainable Poultry Production.” Livestock Science 286: 105525. 10.1016/j.livsci.2024.105525.

[jpn70012-bib-0020] Mountzouris, K. C. , V. V. Paraskeuas , and K. Fegeros . 2020. “Priming of Intestinal Cytoprotective Genes and Antioxidant Capacity by Dietary Phytogenic Inclusion in Broilers.” Animal Nutrition 6: 305–312. 10.1016/j.aninu.2020.04.003. 33005764 PMC7503066

[jpn70012-bib-0021] Mountzouris, K. C. , V. V. Paraskeuas , and E. Griela . 2022. “Adaptive Poultry Gut Capacity to Resist Oxidative Stress.” In Gut Microbiota, Immunity, and Health in Production Animals M. H. Kogut and G. Zhang , 243–262. Springer Nature.

[jpn70012-bib-0022] Paraskeuas, V. , and K. C. Mountzouris . 2019. “Broiler Gut Microbiota and Expressions of Gut Barrier Genes Affected by Cereal Type and Phytogenic Inclusion.” Animal Nutrition 5: 22–31. 10.1016/j.aninu.2018.11.002. 30899806 PMC6407073

[jpn70012-bib-0023] Paraskeuas, V. , A. Pastor , T. Steiner , and K. C. Mountzouris . 2024. “Effects of a Dietary Isoquinoline Alkaloids Blend on Gut Antioxidant Capacity and Gut Barrier of Young Broilers.” Poultry Science 103: 103654. 10.1016/j.psj.2024.103654. PMC11067758 38537403

[jpn70012-bib-0024] Pfaffl, M. W. 2001. “A New Mathematical Model for Relative Quantification in Real‐Time RT‐PCR.” Nucleic Acids Research 29: 45e. 10.1093/nar/29.9.e45. PMC55695 11328886

[jpn70012-bib-0025] Pham, V. H. , W. Abbas , J. Huang , et al. 2022. “Effect of Blending Encapsulated Essential Oils and Organic Acids as an Antibiotic Growth Promoter Alternative on Growth Performance and Intestinal Health in Broilers With Necrotic Enteritis.” Poultry Science 101: 101563. 10.1016/j.psj.2021.101563. PMC8628017 34823183

[jpn70012-bib-0026] Soares, I. , B. L. Belote , E. Santin , G. C. Dal Pont , and M. H. Kogut . 2023. “Morphological Assessment and Biomarkers of Low‐Grade, Chronic Intestinal Inflammation in Production Animals.” Animals: An Open Access Journal From MDPI 12: 3036. 10.3390/ani12213036. PMC9654368 36359160

[jpn70012-bib-0027] Song, B. , J. He , X. Pan , et al. 2023. “Dietary *Macleaya cordata* Extract Supplementation Improves the Growth Performance and Gut Health of Broiler Chickens With Necrotic Enteritis.” Journal of Animal Science and Biotechnology 14: 113. 10.1186/s40104-023-00916-2. 37674220 PMC10483844

[jpn70012-bib-0028] Song, Z. H. , K. Cheng , X. C. Zheng , H. Ahmad , L. L. Zhang , and T. Wang . 2018. “Effects of Dietary Supplementation With Enzymatically Treated *Artemisia annua* on Growth Performance, Intestinal Morphology, Digestive Enzyme Activities, Immunity, and Antioxidant Capacity of Heat‐Stressed Broilers.” Poultry Science 97: 430–437. 10.3382/ps/pex312. 29077887

[jpn70012-bib-0029] Wang, D. , Q. Zhang , Z. Zhang , et al. 2024. “Expression Profile of Toll‑Like Receptors and Cytokines in the Cecal Tonsil of Chickens Challenged With Eimeria Tenella.” Parasitology Research 123: 347. 10.1007/s00436-024-08371-2. 39387973

[jpn70012-bib-0030] Wang, M. , J. Zhang , X. Huang , Y. Liu , and J. Zeng . 2022. “Effects of Dietary *Macleaya cordata* Extract on Growth Performance, Biochemical Indices, and Intestinal Microbiota of Yellow‐Feathered Broilers Subjected to Chronic Heat Stress.” Animals: An Open Access Journal From MDPI 12: 2197. 10.3390/ani12172197. 36077916 PMC9454434

[jpn70012-bib-0031] Wickramasuriya, S. S. , I. Park , K. Lee , et al. 2022. “Role of Physiology, Immunity, Microbiota, and Infectious Diseases in the Gut Health of Poultry.” Vaccines 10: 172. 10.3390/vaccines10020172. 35214631 PMC8875638

[jpn70012-bib-0032] Zhang, L. , X. Wang , S. Huang , Y. Huang , H. Shi , and X. Bai . 2023. “Effects of Dietary Essential Oil Supplementation on Growth Performance, Carcass Yield, Meat Quality, and Intestinal Tight Junctions of Broilers With or Without Eimeria Challenge.” Poultry Science 102: 102874. 10.1016/j.psj.2023.102874. PMC10339057 37406442

